# Sudden emergence of a *Neisseria gonorrhoeae* clade with reduced susceptibility to extended-spectrum cephalosporins, Norway

**DOI:** 10.1099/mgen.0.000480

**Published:** 2020-11-17

**Authors:** Magnus N. Osnes, Xavier Didelot, Jolinda de Korne-Elenbaas, Kristian Alfsnes, Ola B. Brynildsrud, Gaute Syversen, Øivind Jul Nilsen, Birgitte Freiesleben De Blasio, Dominique A. Caugant, Vegard Eldholm

**Affiliations:** ^1^​ Division of Infection Control and Environmental Health, Norwegian Institute of Public Health, Oslo, Norway; ^2^​ School of Life Sciences and Department of Statistics, University of Warwick, Coventry, UK; ^3^​ Department of Infectious Diseases, Public Health Service Amsterdam, Amsterdam, The Netherlands; ^4^​ Department of Microbiology, Oslo University Hospital Ullevål, Oslo, Norway; ^5^​ Oslo Centre for Biostatistics and Epidemiology, Department of Biostatistics, Institute of Basic Medical Sciences, University of Oslo, Oslo, Norway; ^6^​ Department of Community Medicine, Faculty of Medicine, University of Oslo, Oslo, Norway; ^7^​ AMR Centre, Norwegian Institute of Public Health, Oslo, Norway

**Keywords:** *Neisseria gonorrhoeae*, disease outbreaks, phylogeography, genomic epidemiology, antimicrobial resistance, transmission

## Abstract

*
Neisseria gonorrhoeae* multilocus sequence type (ST)-7827 emerged in a dramatic fashion in Norway in the period 2016–2018. Here, we aim to shed light on the provenance and expansion of this ST. ST-7827 was found to be polyphyletic, but the majority of members belonged to a monophyletic clade we termed PopPUNK cluster 7827 (PC-7827). In Norway, both PC-7827 and ST-7827 isolates were almost exclusively isolated from men. Phylogeographical analyses demonstrated an Asian origin of the genogroup, with multiple inferred exports to Europe and the USA. The genogroup was uniformly resistant to fluoroquinolones, and associated with reduced susceptibility to both azithromycin and the extended-spectrum cephalosporins (ESCs) cefixime and ceftriaxone. From a genetic background including the *penA* allele 13.001, associated with reduced ESC susceptibility, we identified repeated events of acquisition of *porB* alleles associated with further reduction in ceftriaxone susceptibility. Transmission of the strain was significantly reduced in Norway in 2019, but our results indicate the existence of a recently established global reservoir. The worrisome drug-resistance profile and rapid emergence of PC-7827 calls for close monitoring of the situation.

## Data Summary

Accession numbers for all sequence reads included in the study are available as supplementary material, including any meta-info used. Norwegian genomic short-read sequences are available under ENA (European Nucleotide Archive) study accession number PRJEB32435. Epidemiological data (not linkable to individual clinical isolates) were retrieved from the Norwegian Surveillance System for Communicable Diseases (MSIS): http://www.msis.no/.

**Impact Statement**

Gonorrhoea is a sexually transmitted disease caused by the bacterium *Neisseria gonorrhoeae *. Over the last decade, the incidence of gonorrhoea has increased dramatically in parts of the world, including Europe. On top of this, the bacterium has historically evolved resistance to all the drugs used to treat the infection. Currently, dual treatment with the macrolide azithromycin and the extended-spectrum cephalosporin ceftriaxone constitutes the recommended therapy in most countries. Reduced susceptibility to azithromycin is already widespread, whereas ceftriaxone remains effective against the vast majority of infections. Ceftriaxone, thus, represents the last near-universally effective drug for treating gonorrhoea. During 2017 and 2018, the incidence of a ‘new’ *N. gonorrhoeae* strain increased manyfold in Norway and became one of the most common causes of gonorrhoea. The strain exhibited reduced susceptibility to both azithromycin and ceftriaxone, and was mainly recovered from men. Our analyses, relying on gonococcal whole-genome sequences from Norway and abroad, point to an unreported global reservoir of the strain. Transmission of the strain was to a large extent curbed in Norway in 2019, but new introductions to the country are expected. Cases of ‘untreatable’ gonorrhoea have been reported, but are still exceedingly rare. The efficient spread of a new strain with reduced susceptibility to key drugs is worrying and the situation should be monitored closely.

## Introduction

*Neisseria gonorrhoeae* represents an increasing burden on health [1]. Globally, the pathogen has acquired resistance to all drugs recommended for therapy [2]. As a result of the rapid acquisition of antibiotic resistance and the accompanying increased risk of treatment failure, the World Health Organization in 2017 listed *N. gonorrhoeae* as a high-priority pathogen, on which research and drug development should focus [3].

The incidence of gonorrhoea is increasing rapidly in Norway. From 1995 to 2010, the number of cases fluctuated between 150 and 300 cases per year, but has since followed a steep upward trajectory, reaching 1704 reported cases in 2019 [4]. Of these cases, 78 % were men and 58 % were infected following homosexual sex [4].

Gonococci are efficient colonizers of mucosae and can infect, in addition to the urethrae, both the pharynx and rectum. In men, urethral infections are mostly symptomatic, and affected individuals tend to seek treatment rapidly, whereas pharyngeal and rectal infections often are asymptomatic and may go undetected for a longer period [5]. In women, cervicitis is the most common manifestation, and infections are often asymptomatic. Thus, it is possible that different infection sites play differential roles in the epidemic process.

Genome-based analyses of geographical dispersal and person-to-person transmission have the potential to uncover unobserved drivers of an epidemic. Previously, such methods have been used to determine time periods for which an outbreak has been poorly sampled [6], declare the end of an outbreak [7], estimate the geographical origin of extant gonorrhoea [8] and assess the importance of importation for transmission sustenance [9]. However, obtaining representative samples is often a challenge when reconstructing phylogeographical and transmission histories.

In the period 2016–2018, there was a sudden surge in cases belonging to the hitherto undescribed sequence type (ST)-7827 among men in Norway. The number of cultured cases belonging to the ST rose from 7 (1.8 %) in 2016 to 146 (19.8 %) in 2018. Here, we apply genome-based analyses to investigate the sudden emergence of ST-7827.

## Methods

### Clinical isolates

The Norwegian Institute of Public Health hosts the National Reference Laboratory for Gonorrhoea and receives all cultured cases from Norway. Whole-genome sequencing has been performed routinely since 2016. Multilocus sequence typing (MLST) is performed *in silico* [10] against the pubMLST database [11] to determine STs. In the period 2016–2018, 159 out of 1791 isolates were found to belong to ST-7827. Sequence data are available under ENA (European Nucleotide Archive) study accession number PRJEB32435. Beyond the study period, MLST results were retrieved from our strain collection for the year 2019.

Metadata attached to available large genome datasets uploaded to the National Center for Biotechnology Information short-read archive (https://www.ncbi.nlm.nih.gov/sra) were screened to identify additional ST-7827 isolates. For datasets where this information was lacking, we downloaded and screened the sequence data *in silico* against the pubMLST database to determine the ST. We did not identify any ST-7827 members among recently published sequences from Japan [12], the USA [13] or a global gonorrhoea genomic study [8]. However, in a recent study from China, 74 out of 435 isolates belonged to ST-7827 [14], and these were included in the current study.

Since ST classifications are imperfect for resolving the *N. gonorrhoeae* population structure [15], available Norwegian genomes from the period 2016–2018 (*n*=1659) in addition to 2093 genomes from the Chinese study (accession no. PRJNA431691) were clustered based on genome assemblies using PopPUNK [16]. Subsequently, we annotated the clustering-derived neighbour-joining tree with ST classifications. This revealed that the vast majority of genomes clustered in a monophyletic clade, which we termed PopPUNK cluster 7827 (PC-7827). However, this clade also included a few non-ST-7827 members, and a small cluster of ST-7827 genomes fell outside this clade. That is, ST-7827 is not monophyletic as a whole (Fig. S1, available with the online version of this article).

The monophyletic PC-7827 clade was mainly made up of ST-7827 isolates, but also included the STs 1904, 13143 and 13489, in addition to one or two isolates each from STs 1583 (*n*=1), 7363 (*n*=1), 8118 (*n*=2), 9304 (*n*=1), 12 971 (*n*=1) and 12 974 (*n*=2). Based on this information, we screened PathogenWatch [17] and the PubMLST database [11] for all the above STs, ignoring ST-1583 and ST-7363, which are relatively common STs globally and which were only found once each in our screen. In total, 410 isolates matched the inclusion criteria (Chinese and Norwegian genomes identified as PC-7827 and/or ST-7827 plus PathogenWatch and PubMLST genomes belonging to STs 7827, 1904, 13143, 13489, 8118, 9304, 12 971 and 12 974), of which 370 belonged to PC-7827 and 344 carried the ST-7827 MLST definition. Out of 186 Norwegian PC-7827 isolates, 180 were isolated from men, whereas for the non-Norwegian isolates with available information, all 52 isolates were recovered from men. Information on all isolates is available in an Excel file (Table S1).

### Genome analyses

Raw Illumina reads were assembled *de novo* using SPAdes [18] in 'careful' mode. The assemblies were further filtered to remove contigs with a kmer-coverage <3 and length<500 nucleotides. To generate a high-quality reference genome for comparative genomic analyses, long-read data were generated on the Oxford Nanopore GridION platform for one of the Norwegian isolates (641 189). DNA was extracted and sequences generated as described previously [19]. For assembly, we employed a hybrid approach implemented in Unicycler v0.4.7 [20], leveraging both long Oxford Nanopore reads and short accurate Illumina reads. This resulted in a resolved circular genome of 2 215 865 bp plus a 4207 bp plasmid.

Parsnp [21] was used to align the short-read assemblies to the closed reference genome (excluding the plasmid). A whole-genome multi-fasta retaining the reference nucleotide position for all sites by filling all non-core regions with reference nucleotides was generated with an in-house script (https://github.com/krepsen/parsnp2fasta). Finally, Gubbins [22] was employed to identify recombination tracts in the whole-genome alignments and a recombination-corrected phylogeny was generated as per default by Gubbins employing a genome belonging to ST-1901 as the outgroup.

### Analyses of antibiotic susceptibility

Minimum inhibitory concentration (MIC) data were available for a handful of the isolates downloaded from PubMLST and PathogenWatch. For the Norwegian isolates, full MIC profiles were available for all isolates from 2016 to 2017, and about 80 % of 2018 isolates. Throughout the text, we refer to the European Committee on Antimicrobial Susceptibility Testing (EUCAST) [23] when breakpoints are discussed (resistance breakpoints: cefixime and ceftriaxone >0.125 µg ml^−1^; ciprofloxacin >0.06 µg ml^−1^). Until recently, an azithromycin MIC ≥0.5 µg ml^−1^ was considered as intermediately resistant by EUCAST, whereas the latest guideline refrains from defining breakpoints. None of the isolates for which MIC data were available reached the epidemiological cut-off value of >1 µg ml^−1^ [24]. Here, we use the term ‘reduced susceptibility’ on isolates with azithromycin MICs of ≥0.25 µg ml^−1^ and cefixime/ceftriaxone MICs of ≥0.064 µg ml^−1^.

NG-STAR types were assigned using PathogenWatch [17]. To annotate alleles relevant for antibiotic resistance, genome assemblies were aligned against the *penA* (NEIS1753), 23S (NG_23S), *gyrA* (NG_gyrA), *parC* (NG_parC) and *porB* (NG_porB) allele databases downloaded from PubMLST [11], using blast [25]. For 23S, the four best hits were retained for each blast search to ensure that mixed alleles were detected if present. When available, the NG-STAR [26] allele nomenclature was used. Results from phenotypic and *in silico* predicted resistance are available in Table S1.

### Additional phylogenetic analyses and transmission modelling

Phylogeographical inferences were made using stochastic character mapping [27] implemented in the phytools R package [28]. To account for phylogenetic uncertainty, we constructed a set of 100 bootstrap trees with PhyML [29] using the 3741 polymorphic sites estimated to be non-recombinant according to Gubbins. For each of the bootstrap trees, we simulated 10 stochastic character mappings, giving us a posterior set of 1000 geographically mapped phylogenetic trees, which was summarized on a maximum-likelihood tree from PhyML. The full phylogeographical phylogeny is included in Fig. S2.

The Gubbins output was used directly as input for BactDating [30] to perform root-to-tip regression and temporal analyses (Figs S3–S5). TransPhylo [6] was used to estimate transmission trees. These analyses were performed on ST-7827 clusters 1 and 3 only, as they were performed before the inclusion of PC-7827 members belonging to other STs (most importantly, ST-13489). As the analyses are extremely computer-intensive, they were not repeated, but we note that this would only affect the deeper branches of the transmission trees, and are highly unlikely to affect our inferences. For the generation time distribution (time from being infected to infecting others), we considered two gamma distributions, one which was estimated in an individual-based modelling study from a community of men who have sex with men (MSM) [31] – ‘prior 1’ with shape 0.57 and scale 0.30, and a distribution mimicking prior 1 but penalizing transmissions in the incubation phase – ‘prior 2’ with shape 1.20 and scale 0.14 (Fig. S6). The sampling distribution (time between infection and sampling) was set equal to the generation time distribution. Based on the discrepancy between the number of reported cases and cultured cases over time, we know that about 55 % of cases are lost due to failed culturing. In addition, we must assume that some, particularly asymptomatic cases, go undiagnosed. Yet, in Norway, this fraction is expected to be modest as high-risk individuals are screened frequently and contact tracing is performed on all cases. Based on the above, we fixed sampling densities in TransPhylo to the fractions 0.2, 0.3, 0.4 and 0.5 of the number of cases. Sensitivity testing indicated that the modelling output was not overly sensitive to the choice of generation time and sampling density priors (Figs S7–S9). In the end, we used the results generated with a sampling density of 0.4 and prior 2 for the generation time.

From the estimated transmission trees, we extracted the posterior generation times, the number of secondary infections caused by primary infections, the pairwise individual-to-individual probability of direct transmissions and the mean number of intermediate carriers between each pair of patients. To investigate whether infections of the urethra, rectum and pharynx differed in their propensity to transmit to secondary cases, we stratified the posterior generation times and the number of secondary infections, on different infection sites. The methods applied in this section are described in detail in Supplementary Methods.

## Results

### Population genomic characteristics of ST-7827

**Fig. 1.**
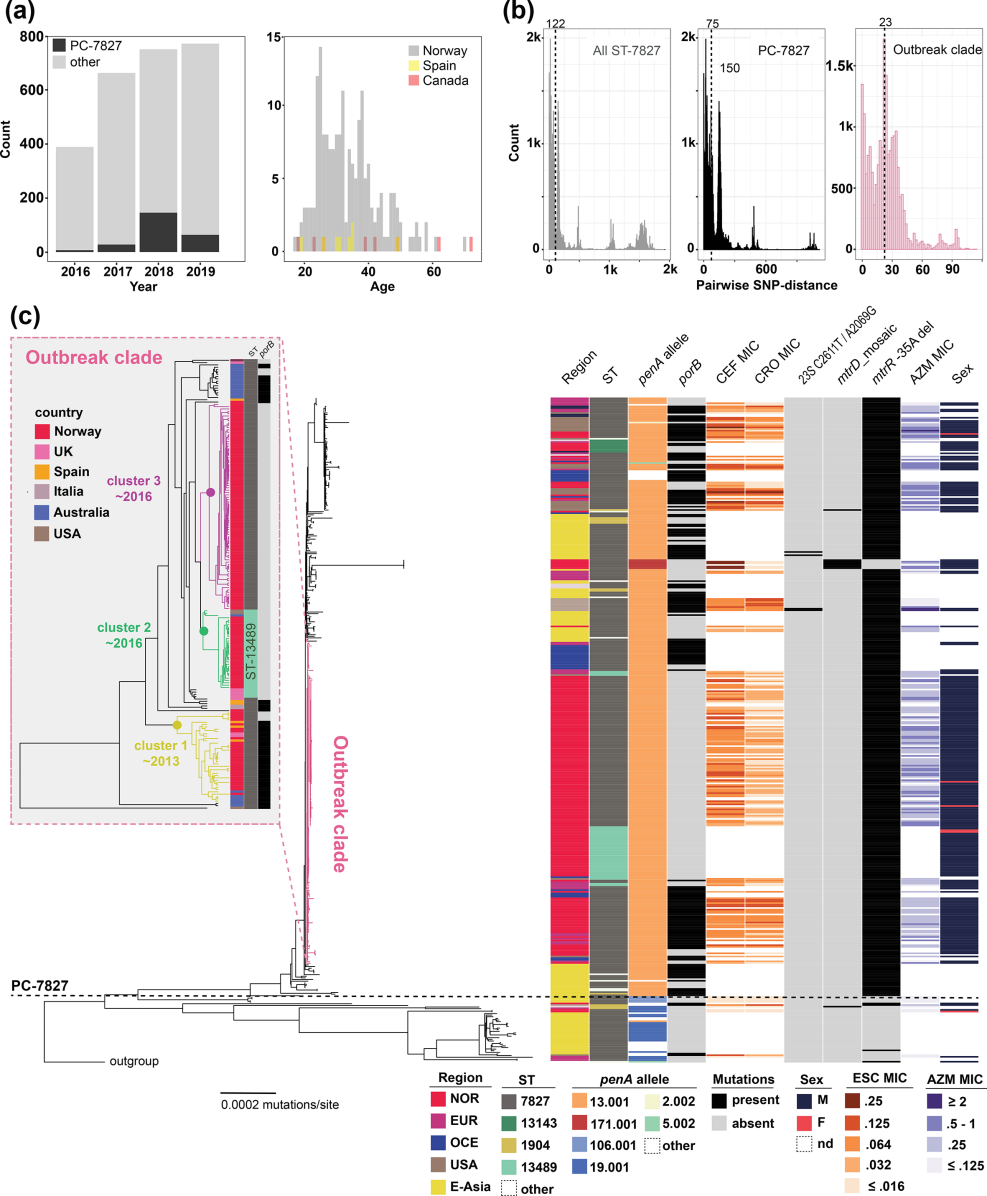
Overview of PC-7827 incidence and genomic properties. (a) Incidence of PC-7827 among culture-positive *N. gonorrhoeae* cases in relation to the total number of cultured cases during 2016–2019 in Norway (left). Age distribution of ST-7827 cases in Norway, Spain and Canada (right). (b) Pairwise median SNP-distances within ST-7827, PC-7827 and the recently emerged outbreak clade. Numbers above the dotted lines refer to median pairwise SNP-distance. (c) Annotated recombination-corrected genome-based phylogeny of 410 ST-7827 and PC-7827 genomes. The horizontal dotted line highlights the split between PC-7827 and unrelated ST-7827 genomes. An ST-1901 member was used as an outgroup. NOR, Norway; EUR, Europe; OCE, Oceania; E-Asia, East Asia; CEF, cefixime; CRO, ceftriaxone; AZM, azithromycin. *porB* specifies the presence of both mutations G120K and A121. The inset figure represents a zoomed-in dated phylogeny of the outbreak clade.

Genome-clustering of the Norwegian as well as a large Chinese dataset revealed that ST-7827 was polyphyletic (see Fig. S1 and Methods). The majority of isolates, however, belonged to a large monophyletic clade we termed PC-7827. This clade also included 65 isolates assigned to other STs (Fig. 1). Screening of available published genomes (see Methods) resulted in a total of 370 genomes belonging to PC-7827, of which 184 were sampled in Norway during the study period January 2016 to December 2018. The rest of the isolates were from across Europe, East Asia, Oceania and North America, mainly from China (*n*=87), Australia (*n*=35), the USA (*n*=28) and the UK (*n*=28). The oldest ST-7827 specimen was isolated in Canada in 1997, but all of the PC-7827 isolates were sampled between 2011 and 2018. PC-7827 isolates before 2015 were largely from East Asia (74 %), which was true for only 2 % of isolates from the period 2015–2018. Among the Norwegian cases, 97 % were men, and 90 % resided in the greater Oslo area. In Norway, the proportion of cultured isolates belonging to PC-7827 increased abruptly from 1.8 % in 2016 to 19.8 % in 2018, with most cases being from adult men between 25 and 50 years old (Fig. 1a). Demographic data were also available for a total of 52 Spanish and Canadian isolates, all of which were from men whose age-distribution mirrored the one in Norway, indicating transmission in similarly structured MSM networks. Interestingly, in 2019, the transmission of the ST seems to have been significantly reduced in Norway, accounting for only 8.3 % of cultured cases.

More than half (199 out of 370) of the PC-7827 isolates were part of a short-branched outbreak clade (median pairwise SNP-distance of 23) (Fig. 1b), bearing the hallmarks of recent expansion outside East Asia. Of the isolates belonging to the outbreak clade, 157 were from Norway, the rest from Australia, the USA and other European countries.

Out of 370 PC-7827 isolates, 362 harboured the *gyrA* S91F mutation, and the vast majority of isolates carried additional *gyrA* and *parC* mutations. All but one strain tested were phenotypically resistant to ciprofloxacin (Table S1), which is in line with early observations of ST-7827 [32]. The *mtrR* promoter deletion −35A was universally present in the genogroup, except for a cluster where the mutation had reverted and which carried a mosaic *mtrD*. Nearly all tested isolates had azithromycin MICs between 0.25 and 1 µg ml^−1^ (Fig. 1c).

Within PC-7827, the *penA* allele 13.001 was by far the most common (Fig. 1c). The *penA* 13.001 allele is non-mosaic but carries an A501V mutation and has been shown to be associated with moderately elevated extended-spectrum cephalosporin (ESC) MICs of ≥0.03 µg ml^−1^ [33, 34]. Across the genogroup, we also identified repeated acquisition of *porB* alleles carrying G120K and A121D mutations. These mutations have previously been associated with elevated ESC MICs in combination with *penA* [33]. As can be seen in Fig. 1(c), acquisition of the mutated *porB* was associated with a reduction in ceftriaxone susceptibility, with the strains carrying it typically having MICs of 0.064–0.125 µg ml^−1^. The annotated phylogeny suggests that acquisitions of these *porB* alleles have occurred repeatedly, across world regions. A small cluster of cefixime-resistant isolates, all isolated in Europe, had acquired the mosaic *penA* 171.001, which is closely related to the 10.001 allele (Fig. 1c). The 10.001 allele and its variants have previously been shown to be associated with cefixime resistance, but not ceftriaxone [12], which fits our observations well. The 171.001 allele harbours two mutations (P538S and T549A) relative to 10.001, but is otherwise identical.

### Spatial and temporal inferences

**Fig. 2.**
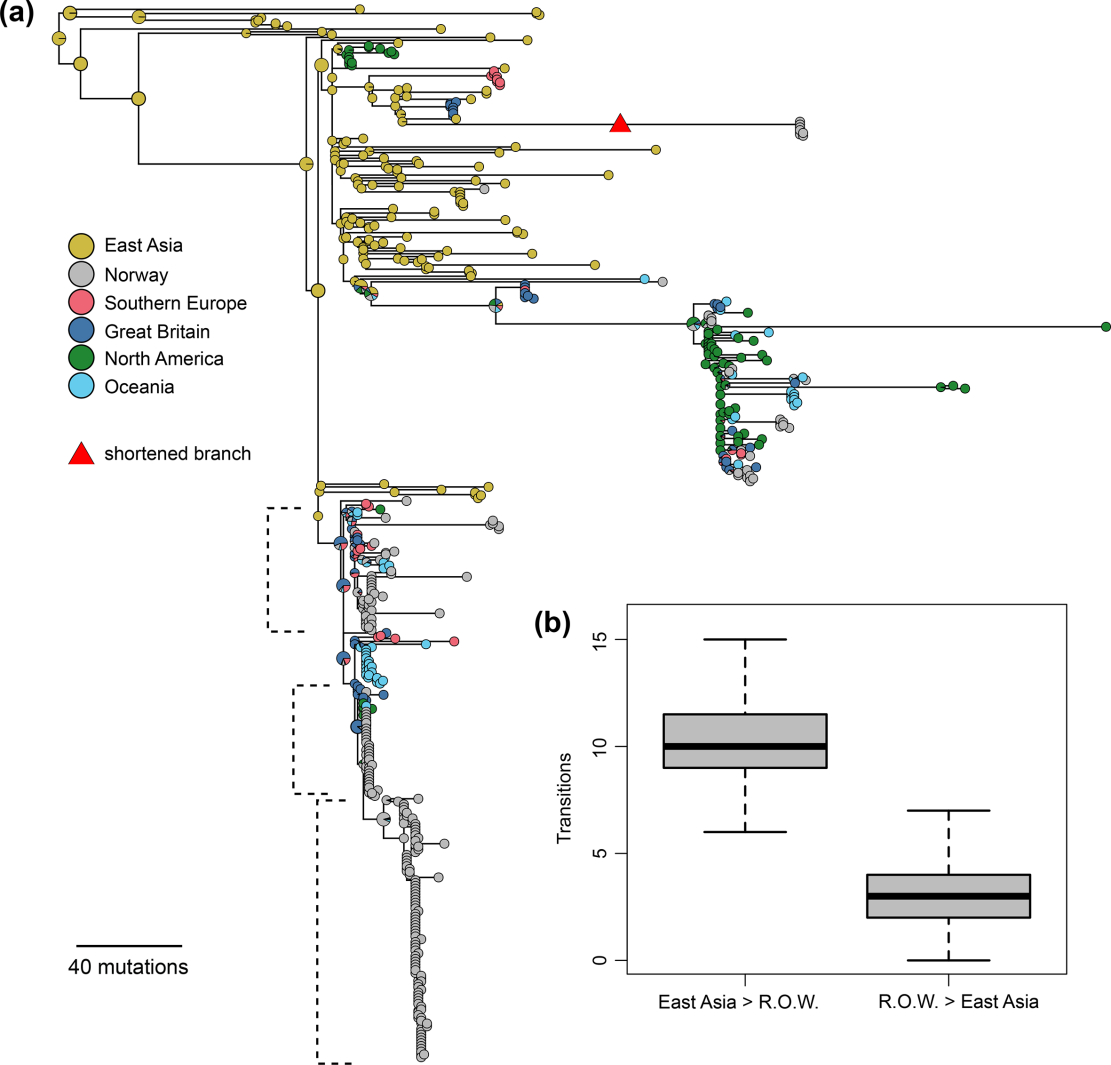
Phylogeographical inference. (a) Estimated geographical locations of PC-7827 mapped on a midpoint-rooted maximum-likelihood phylogeny from PhyML. To make the tree easier to read, some of the deeper branches were removed and one long branch was shortened (indicated by a red triangle). The full image is included in Fig S2. (b) Box plot summarizing the estimated number of transition events between East Asia and the rest of the world (RoW) over 1000 simmap simulations.

From the phylogeny, it seemed clear that the ancestor of PC-7827 emerged in Asia. To generate a more detailed understanding of the geographical origins and spread of the genogroup, we employed simmap [27] to reconstruct the phylogeographical history of the genogroup. A maximum-likelihood tree with the estimated geographical states of the ancestral tree is shown in Fig. 2(a), and the estimated number of exports from Asia to the rest of the world (and vice versa) is shown in Fig. 2(b). Together, these indicate that PC-7827 was exported from East Asia on multiple separate occasions.

**Fig. 3.**
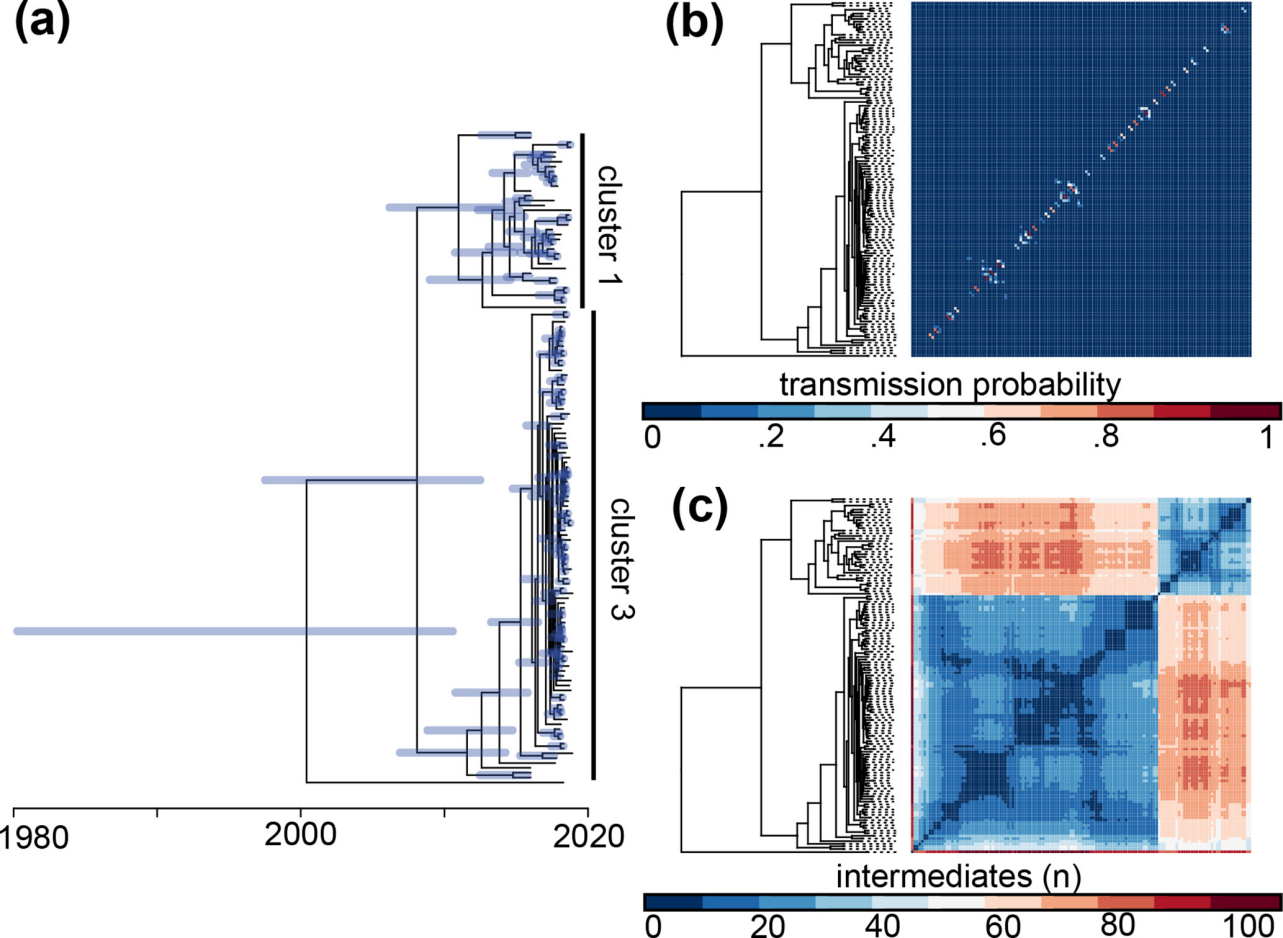
Reconstruction of temporal evolution and transmission patterns in the European outbreak. (a) Dated phylogeny of the European outbreak generated with BactDating. Node bars indicate 95 % highest posterior density intervals. (b) Heatmap ordered after the phylogeny showing the posterior probability of direct transmissions between each pair of patients, calculated as the fraction of the transmission trees where the pair is directly connected. (c) Similar heatmap showing the mean number of intermediate individuals between each pair of patients over all the transmission trees.

Subsequently, a dated phylogeny of the outbreak clade was generated using BactDating (Fig. 3a, and see Supplementary Methods). The mean estimated substitution rate in the non-recombining regions of the genome was 2.2×10^−6^ per site per year (confidence interval 1.5×10^−6^ to 3.2×10^−6^), which is very similar to previous estimates of *N. gonorrhoeae* mutation rates [8, 13, 35]. The outbreak clade contained three distinct sub-clusters dominated by Norwegian isolates (clusters 1–3) with a time of the most recent common ancestor (TMRCA) around 2008. The TMRCA of each of the three transmission clusters were inferred to be around 2013 for cluster 1 and 2016 for clusters 2 and 3 (inset image in Fig. 1c). Intriguingly, despite the restricted diversity within the entire outbreak clade, illustrated by a median pairwise SNP-distance of 23, isolates belonging to cluster 2 were characterized by an allelic replacement resulting in a changed MLST profile (ST-13489).

The phylogeographical analyses (Fig. 2a) revealed that the ancestor of the outbreak clade was introduced to Europe from Asia, as the geographical location is mapped to Asia in their common basal branches. Whereas clusters 2 and 3 were geographically restricted to Norway, and likely introduced by single importation events (Fig. S10), cluster 1 consisted of multiple geographical locations intermixed between the Norwegian samples, indicating that this part of the outbreak may have been sustained by multiple importation events.

### Transmission analyses

In order to investigate transmission dynamics in more detail, we reconstructed genome-based transmission trees using TransPhylo [6], with the dated phylogeny made using BactDating as input. These analyses were performed on outbreak clade members assigned to ST-7827, which in practical terms translates to clusters 1 and 3. For the outbreak as a whole, the reproduction number was estimated to be *R*=1.20 (confidence interval 1.10, 1.31), and this was not sensitive to different assumptions on sampling densities (see Fig. S9 and Supplementary Methods). The estimated probabilities of direct transmission between each pair of patients are shown in Fig. 3(b). In total, there were 24 pairs with a direct transmission probability higher than 50 % (cumulatively 33 with a probability higher than 40 % and 42 higher than 30 %). This indicates that, when the genomic data are restricted to successfully cultured isolates, we are not able to fully resolve the transmission chains, since for most patients there is a higher probability that their infector is unobserved than among the sampled individuals. The mean number of intermediate carriers is shown in Fig. 3(c).

Although intermediate unsampled carriers were inferred between most pairs of patients, groups of patients with few intermediates between them are clearly visible (note the blue squares in the heatmap, which indicates very few intermediate carriers for the group). When excluding the ‘Norway-only’ transmission clusters (clusters 2 and 3), the majority of the outbreak clade members carry a *porB* allele (G120K and A121D) associated with elevated ceftriaxone MICs (Fig. 1c). This finding is consistent with ongoing circulation of PC-7827 members in Europe, the USA and Australia with a worrisome drug-susceptibility profile.

Next, we investigated whether infections affecting different body sites differed in their transmissibility. The estimated number of transmissions caused by primary infections across different infection sites is shown in Fig. S11(a), and for each patient individually in Fig. S12. Fig. S11(b) is similarly stratified with the posterior generation times plotted along with the prior distribution. None of the analyses showed any clear differences in transmission propensity between infections at different body sites.

## Discussion

ST-7827 emerged as one of the most frequent *N. gonorrhoeae* STs in Norway over a very short period of time (2016–2018), almost exclusively infecting men. The ST was found to be polyphyletic, but the majority of isolates belong to a monophyletic clade we termed PC-7827. We find that PC-7827 originated in East Asia, from where it has been exported to the rest of the world on multiple occasions. The majority of PC-7827 isolates in Norway belonged to an outbreak clade representing three discrete transmission clusters. The oldest transmission cluster originated around 2013 and isolates belonging to it were identified across Europe, the USA and Australia. The other two transmission clusters were restricted to Norway, and were found to have emerged around 2016, which aligns perfectly with incidence data. Importantly, within the outbreak clade, the internationally dispersed cluster 1 is associated with decreased susceptibility to ceftriaxone due to the acquisition of a *porB* allele carrying G120K and A121D mutations.

Due to a relative scarcity of recently sampled genomes globally, it is difficult to assess how widespread the genogroup actually is. Yet the degree of international dissemination combined with multiple inferred imports to Norway suggest that the PC-7827 outbreak clade is well established in the ‘Western world’. We recently learnt that cases of ST-7827 have been observed frequently in the Netherlands since 2017 (Alje van Dam, personal communication), which supports this notion.

Even though we do not have information on the sexual orientation of patients, the gender distribution of cases, both in Norway (97 % men), Spain (100 % men) and Canada (100 % men), is highly suggestive of transmission in MSM networks. Genome-based transmission analyses did not identify any differences in transmissibility between infections affecting different body sites (urethrae, pharynx, rectum). This finding is somewhat surprising as these infections are generally quite different in terms of symptoms and time to clearance [5], and extragenital infections have been suggested to constitute disease reservoirs of particular importance [36]. Recent studies have also highlighted the likely importance of pharyngeal infections in the transmission of gonorrhoea (see, for example, the report by Fairley *et al*. [37]). The fact that we did not identify any epidemiological differences between infections affecting different body sites does indeed suggest that rectal and pharyngeal infections are no less important than genital infections for gonorrhoea transmission, but our results do not support an outsized importance of extragenital relative to genital infections in the current setting.

The antibiotic susceptibility profile of PC-7827, exhibiting uniform resistance to ciprofloxacin and reduced susceptibility to both the first-line drugs ceftriaxone and azithromycin, is concerning. We demonstrate that a *porB* allele significantly reducing ceftriaxone susceptibility has been acquired repeatedly within PC-7827. The establishment of a ‘new’ genogroup with a challenging susceptibility profile should be monitored closely.

PC-7827 has demonstrated an impressive ability to transmit and to become established among MSM over a very short time frame. However, in 2019 the spread of the ST seems to have been partially contained in Norway, probably reflecting the presence of an efficient control programme, encompassing frequent screening of high-risk individuals and contact tracing around all cases.

## Supplementary Data

Supplementary material 1

Supplementary material 2

## References

[1] Wi T, Lahra MM, Ndowa F, Bala M, Dillon J-AR (2017). Antimicrobial resistance in *Neisseria gonorrhoeae*: global surveillance and a call for international collaborative action. PLoS Med.

[2] Unemo M (2015). Current and future antimicrobial treatment of gonorrhoea - the rapidly evolving *Neisseria gonorrhoeae* continues to challenge. BMC Infect Dis.

[3] World Health Organization (2017). *Global Priority List of Antibiotic-Resistant Bacteria to Guide Research, Discovery, and Development of New Antibiotics* (https://www.who.int/medicines/publications/global-priority-list-antibiotic-resistant-bacteria/en/).

[4] MSIS (2020). http://www.msis.no/.

[5] Kent CK, Chaw JK, Wong W, Liska S, Gibson S (2005). Prevalence of rectal, urethral, and pharyngeal chlamydia and gonorrhea detected in 2 clinical settings among men who have sex with men: San Francisco, California, 2003. Clin Infect Dis.

[6] Didelot X, Fraser C, Gardy J, Colijn C (2017). Genomic infectious disease epidemiology in partially sampled and ongoing outbreaks. Mol Biol Evol.

[7] Hatherell H-A, Didelot X, Pollock SL, Tang P, Crisan A (2016). Declaring a tuberculosis outbreak over with genomic epidemiology. Microb Genom.

[8] Sánchez-Busó L, Golparian D, Corander J, Grad YH, Ohnishi M (2019). The impact of antimicrobials on gonococcal evolution. Nat Microbiol.

[9] Ayabina D, Ronning JO, Alfsnes K, Debech N, Brynildsrud OB (2018). Genome-based transmission modelling separates imported tuberculosis from recent transmission within an immigrant population. Microb Genom.

[10] Seemann T (2020). https://github.com/tseemann/mlst.

[11] Jolley KA, Bray JE, Maiden MCJ (2018). Open-access bacterial population genomics: BIGSdb software, the PubMLST.org website and their applications. Wellcome Open Res.

[12] Yahara K, Nakayama S-I, Shimuta K, Lee K-I, Morita M (2018). Genomic surveillance of *Neisseria gonorrhoeae* to investigate the distribution and evolution of antimicrobial-resistance determinants and lineages. Microb Genom.

[13] Grad YH, Kirkcaldy RD, Trees D, Dordel J, Harris SR (2014). Genomic epidemiology of *Neisseria gonorrhoeae* with reduced susceptibility to cefixime in the USA: a retrospective observational study. Lancet Infect Dis.

[14] Peng J-P, Yin Y-P, Chen S-C, Yang J, Dai X-Q (2019). A whole-genome sequencing analysis of *Neisseria gonorrhoeae* isolates in China: an observational study. EClinicalMedicine.

[15] Harrison OB, Cehovin A, Skett J, Jolley KA, Massari P (2020). *Neisseria gonorrhoeae* population genomics: use of the gonococcal core genome to improve surveillance of antimicrobial resistance. J Infect Dis.

[16] Lees JA, Harris SR, Tonkin-Hill G, Gladstone RA, Lo SW (2019). Fast and flexible bacterial genomic epidemiology with PopPUNK. Genome Res.

[17] Wellcome Sanger Institute (2020). https://www.sanger.ac.uk/science/tools/pathogenwatch.

[18] Bankevich A, Nurk S, Antipov D, Gurevich AA, Dvorkin M (2012). SPAdes: a new genome assembly algorithm and its applications to single-cell sequencing. J Comput Biol.

[19] Brynildsrud OB, Eldholm V, Bohlin J, Uadiale K, Obaro S (2018). Acquisition of virulence genes by a carrier strain gave rise to the ongoing epidemics of meningococcal disease in West Africa. Proc Natl Acad Sci USA.

[20] Wick RR, Judd LM, Gorrie CL, Holt KE (2017). Unicycler: resolving bacterial genome assemblies from short and long sequencing reads. PLoS Comput Biol.

[21] Treangen TJ, Ondov BD, Koren S, Phillippy AM (2014). The Harvest suite for rapid core-genome alignment and visualization of thousands of intraspecific microbial genomes. Genome Biol.

[22] Croucher NJ, Page AJ, Connor TR, Delaney AJ, Keane JA (2015). Rapid phylogenetic analysis of large samples of recombinant bacterial whole genome sequences using Gubbins. Nucleic Acids Res.

[23] European Committee on Antimicrobial Susceptibility Testing (2020). *Breakpoint Tables for Interpretation of MICs and Zone Diameters*, version 10.0 (http://www.eucast.org/clinical_breakpoints/).

[24] Kersh EN, Allen V, Ransom E, Schmerer M, Cyr S (2020). Rationale for a *Neisseria gonorrhoeae* susceptible–only interpretive breakpoint for azithromycin. Clin Infect Dis.

[25] Altschul SF, Gish W, Miller W, Myers EW, Lipman DJ (1990). Basic local alignment search tool. J Mol Biol.

[26] Demczuk W, Sidhu S, Unemo M, Whiley DM, Allen VG (2017). *Neisseria gonorrhoeae* sequence typing for antimicrobial resistance, a novel antimicrobial resistance multilocus typing scheme for tracking global dissemination of *N. gonorrhoeae* strains. J Clin Microbiol.

[27] Bollback JP (2006). SIMMAP: stochastic character mapping of discrete traits on phylogenies. BMC Bioinformatics.

[28] Revell LJ (2012). phytools: an R package for phylogenetic comparative biology (and other things). Methods Ecol Evol.

[29] Guindon S, Dufayard J-F, Lefort V, Anisimova M, Hordijk W (2010). New algorithms and methods to estimate maximum-likelihood phylogenies: assessing the performance of PhyML 3.0. Syst Biol.

[30] Didelot X, Croucher NJ, Bentley SD, Harris SR, Wilson DJ (2018). Bayesian inference of ancestral dates on bacterial phylogenetic trees. Nucleic Acids Res.

[31] Whittles LK, White PJ, Didelot X (2019). A dynamic power-law sexual network model of gonorrhoea outbreaks. PLoS Comput Biol.

[32] Alfsnes K, Eldholm V, Olsen AO, Brynildsrud OB, Bohlin J (2020). Genomic epidemiology and population structure of *Neisseria gonorrhoeae* in Norway, 2016–2017. Microb Genom.

[33] Liao M, Gu W-M, Yang Y, Dillon J-AR (2011). Analysis of mutations in multiple loci of *Neisseria gonorrhoeae* isolates reveals effects of PIB, PBP2 and MtrR on reduced susceptibility to ceftriaxone. J Antimicrob Chemother.

[34] Tomberg J, Fedarovich A, Vincent LR, Jerse AE, Unemo M (2017). Alanine 501 mutations in penicillin-binding protein 2 from *Neisseria gonorrhoeae*: structure, mechanism, and effects on cephalosporin resistance and biological fitness. Biochemistry.

[35] De Silva D, Peters J, Cole K, Cole MJ, Cresswell F (2016). Whole-genome sequencing to determine transmission of *Neisseria gonorrhoeae*: an observational study. Lancet Infect Dis.

[36] Valejo Coelho MM, Matos-Pires E, Serrão V, Rodrigues A, Fernandes C (2018). Extragenital gonorrhoea in men who have sex with men: a retrospective study in a STI clinic in Lisbon, Portugal. Acta Med Port.

[37] Fairley CK, Cornelisse VJ, Hocking JS, Chow EPF (2019). Models of gonorrhoea transmission from the mouth and saliva. Lancet Infect Dis.

