# Supplementary material for: Sudden emergence of a Neisseria gonorrhoeae clade with reduced susceptibility to extended-spectrum cephalosporins, Norway
Source: Microb Genom. 2020 Nov 17;6(12):mgen000480. doi: 10.1099/mgen.0.000480 (PMC8116678; doi:10.1099/mgen.0.000480)
Supplement: Supplementary material 1 [file mgen-6-480-s001.pdf]

Appendix 1: Supplementary methods and figures for «*The sudden emergence of a Neisseria gonorrhoeae clade with reduced susceptibility to extended-spectrum cephalosporins, Norway*»

Table of contents

SUPPLEMENTARY METHODS **1**

Temporal analyses and subdivision of the data 1

Phylogeographic analysis 1

Transmission modeling 2

SUPPLEMENTARY FIGURES **3**

Neighbor-joining tree built from PopPUNK-generated genome clustering of Norwegian and Chinese genomes 3

Full phylogeographic mapping 4

Assessment of the temporal signal in the data subsets 5-7

Prior distributions used for the generation time and sampling density in TransPhylo 8

Convergence diagnostics of the TransPhylo MCMC-chains 9

Sensitivity analysis of the TransPhylo estimates 10

Norwegian clusters in the phylogeographic mapping 11

Posterior offspring and generation time distributions 12

Posterior estimates of the individual level number of transmissions 13

References 14

SUPPLEMENTARY METHODS

Temporal analyses and subdivision of the data

For each of the sample subsets PC-7827 and the outbreak clade embedded within, parsnp and Gubbins steps were run independently. The Gubbins output for the outbreak clade was then used as input for BactDating [1] to perform a root-to-tip regression and temporal analyses. BactDating incorporates information on branch-specific recombination rates, and as such is well suited to analyze genetic data from *N. gonorrhoeae* that frequently undergo recombination [2].

To ensure that the temporal estimates in BactDating were not the result of spurious structures in the dataset, we performed 10 tip-date randomizations on the outbreak clade (restricted to isolates matching the ST-7827 multilocus sequence-type) and checked that the posterior 95% credibility interval of the estimated rate did not overlap with the posterior credibility intervals obtained for the randomized data (Fig. S2).

Phylogeographic analysis

To estimate the geographical origin of PC-7827, we used stochastic character mapping [3] implemented in the function make.simmap in the phytools R package [4]. We accounted for phylogenetic uncertainty by constructing a set of 100 bootstrap trees with PhyML [5] using the polymorphic sites estimated to be non-recombinant according to Gubbins. In PhyML, we used the Bayesian Information Criterion (BIC) to select the best substitution model using Smart Model Selection [6]. The Generalised time reversible (GTR) without any decoration had the lowest BIC. For each bootstrap tree, we considered three models for the transition rates between the geographical locations: equal rates (ER), symmetric rates (SYM), and all rates different (ARD). SYM had the lowest Akaike Information Criterion (AIC) in 83 of 100 trees, with a median AIC-difference of -7.9162 from the second-best, which was ARD in all cases. ARD was the best model in the remaining 17 out of the 100 trees. However, for some of the trees SYM had an AIC value more than 300 higher than the ARD model, indicating that it gave a very poor description of these trees. Therefore we used the ARD model when summarizing the results. For each of the bootstrap trees, we ran 10 stochastic character mappings, giving us a posterior set of 1000 geographically mapped phylogenetic trees. The mapped trees were summarized on a consensus tree with node probabilities that were computed as the fraction of mapped trees where nodes were mapped to each location.

Transmission modeling

The Gubbins output was used as input for BactDating [1] to perform root-to-tip regression and temporal analyses. TransPhylo [7] was used to estimate transmission trees for Clusters 1 and 3 (see Fig 1 C). TransPhylo takes a time-dated phylogenetic tree as input and uses information on the generation time of the pathogen, the sampling time distribution, and sampling proportion of individuals with the disease in the population, to produce posterior transmission trees using Markov chain Monte Carlo (MCMC) sampling. For the generation time distribution, we considered two gamma distributions, one which was estimated in an individual-based modeling study from an MSM community [8] - “prior 1” with shape 0,57 and scale 0,3, and a distribution mimicking prior 1 but penalizing transmissions in the incubation phase - “prior 2” with shape 1,2 and scale 0,14 (Fig. S5). The sampling distribution was set equal to the generation time distribution in all cases. Based on the discrepancy between the number of reported cases and cultured cases over time, we know that about 55% of cases are lost due to failed culturing. In addition, we must assume that some, particularly asymptomatic cases, go undiagnosed. Yet, in Norway, this fraction is expected to be moderate as high-risk individuals are screened frequently and contact tracing performed on all cases. Based on this, we fixed sampling densities in TransPhylo to the fractions 0,2, 0,3, 0,4 and 0,5 of the number of cases, which covers the plausible range with a good margin. For each configuration, we ran two MCMC chains from different starting values with 40 million iterations. The number of iterations was thinned down to 20000, and the first half of the iterations were discarded as burn-in. The remaining 10000 iterations in the two chains were combined to a single chain which was used to summarize the results. Convergence diagnostics are shown in Fig. S6 and S7. The output of the transmission modeling, relying on all eight possible combinations of sampling and generation time priors were compared to assess the sensitivity to the choice of priors. By comparing the estimates of the reproductive number, the within-host effective population size (Fig. S8), and the number of inferred direct transmissions we concluded that the results were not sensitive to the choice of priors. We thus chose to rely on the results generated with a sampling density of 0,4 and prior 2 for the generation time.

SUPPLEMENTARY FIGURES

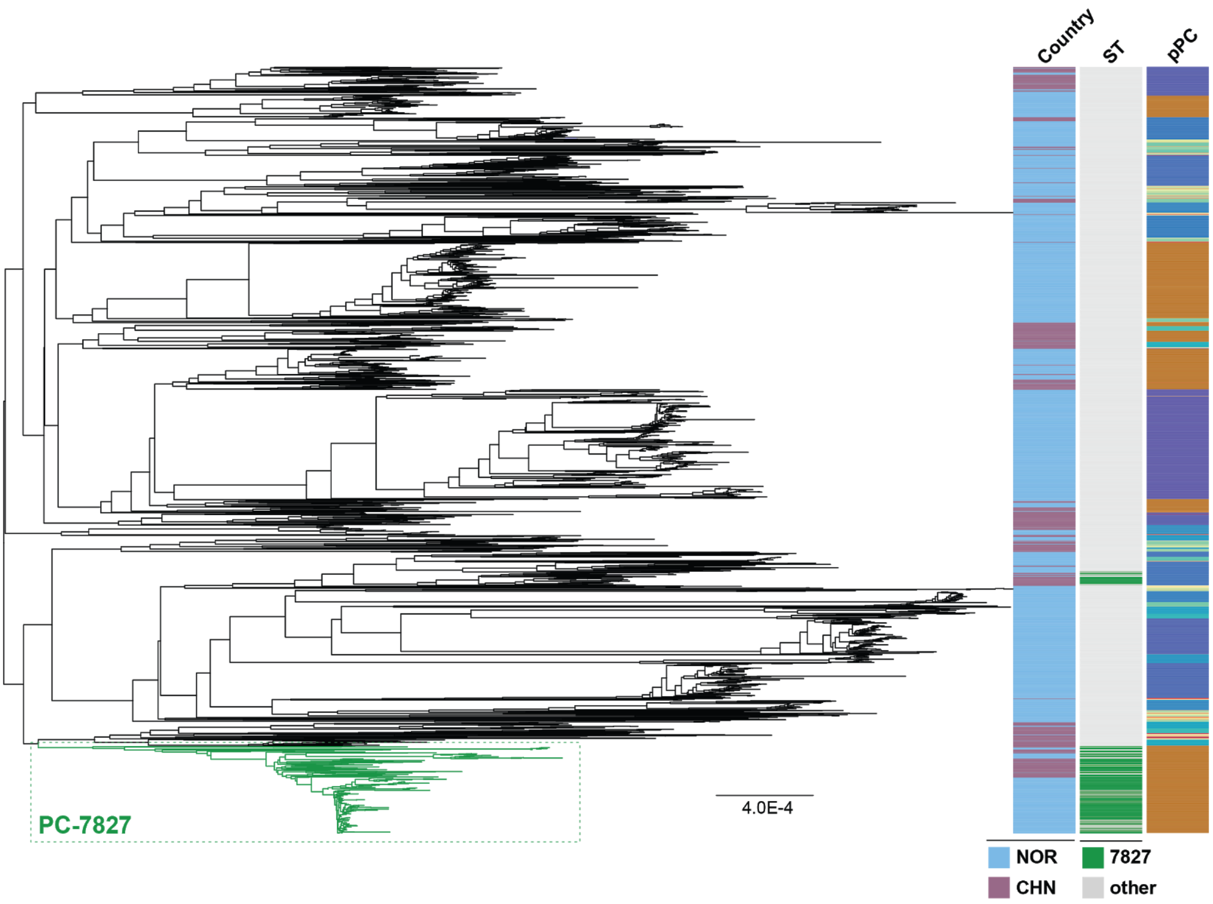

Figure S1. NJ-tree built from PopPUNK-generated genome clustering of Norwegian and Chinese genomes. The monophyletic PC-7827 is highlighted in the tree. A second cluster of ST-7827 not belonging to the monophyletic clade can be identified by the green ST annotation. Identified popPUNK clusters are annotated in the rightmost column.

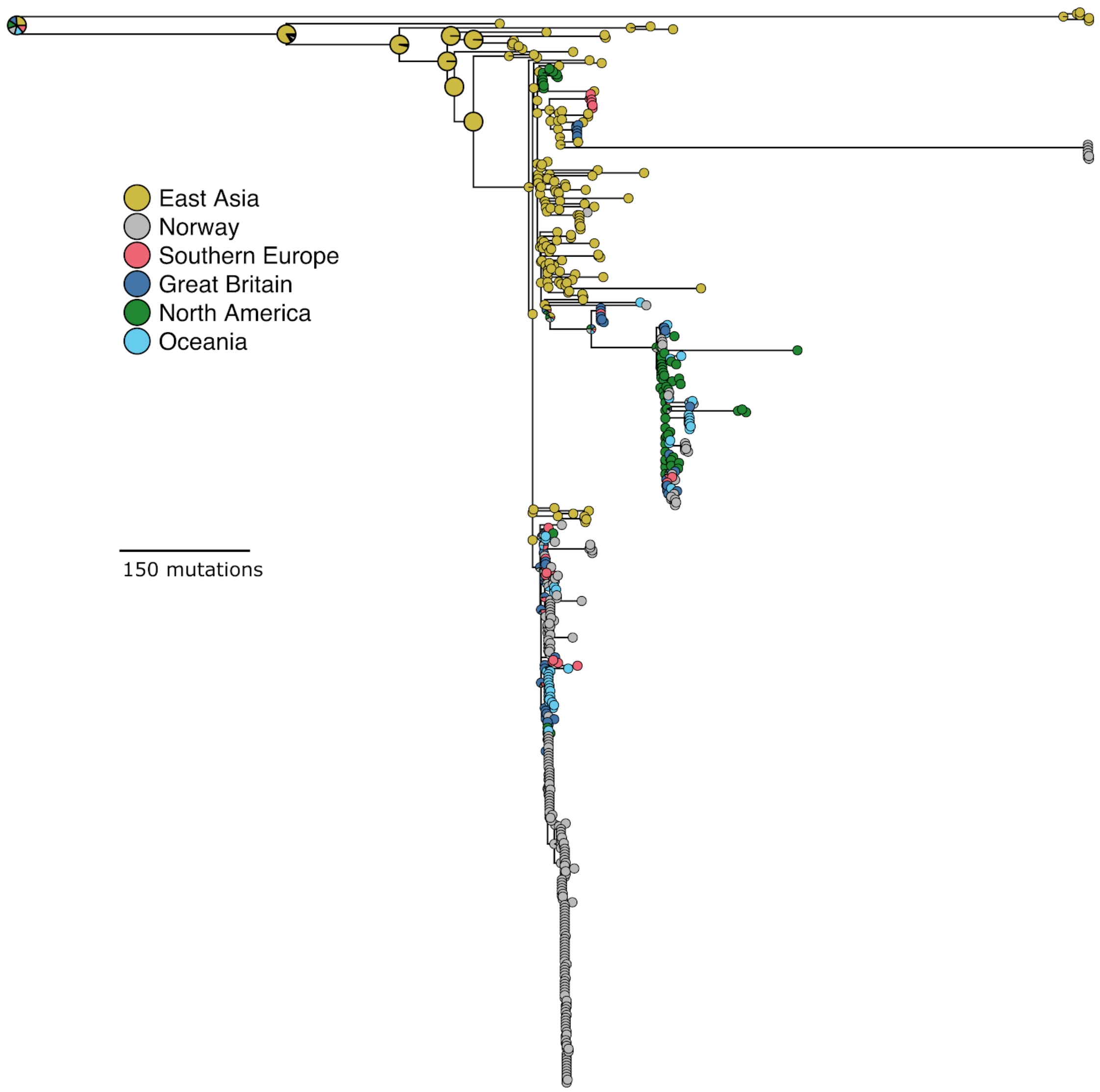

Figure S2. Full phylogeographic mapping. The full tree from the phylogeographic mapping with unaltered branch lengths, including the tips that were removed from Fig. 2 in the main text.

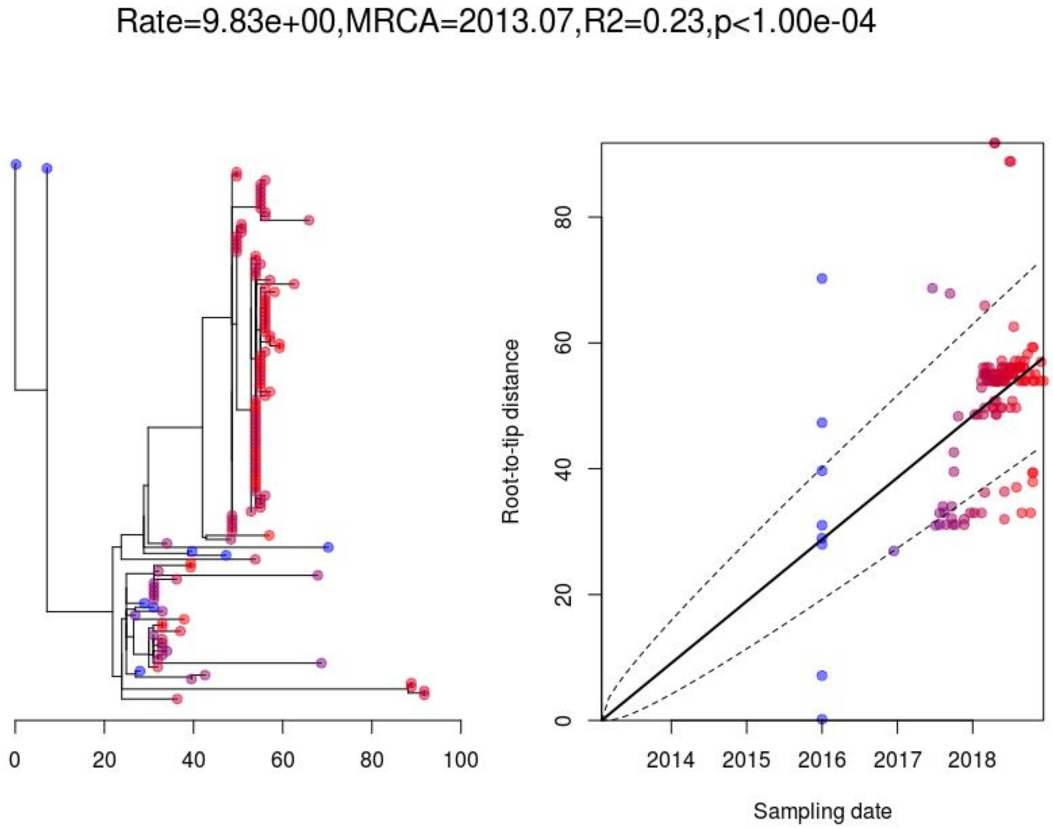

Figure S3. Temporal signal in outbreak clade including all isolates. Regression of the genetic distance on the sampling dates from BactDating.

Rate=7.26e+00,MRCA=2011.13,R2=0.24,p<1.00e−04

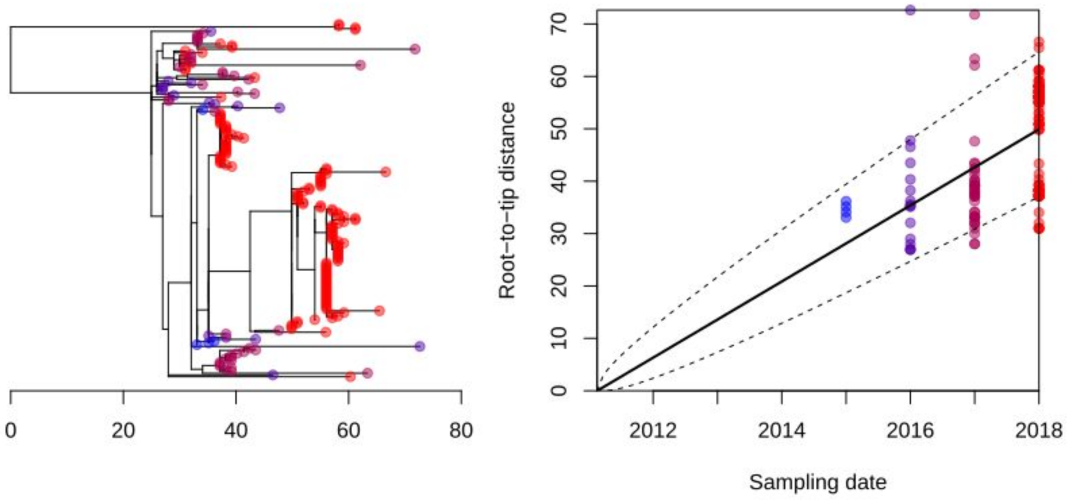

Figure S4. Temporal signal in outbreak clade including only ST-7827. Regression of the genetic distance on the sampling dates from BactDating.

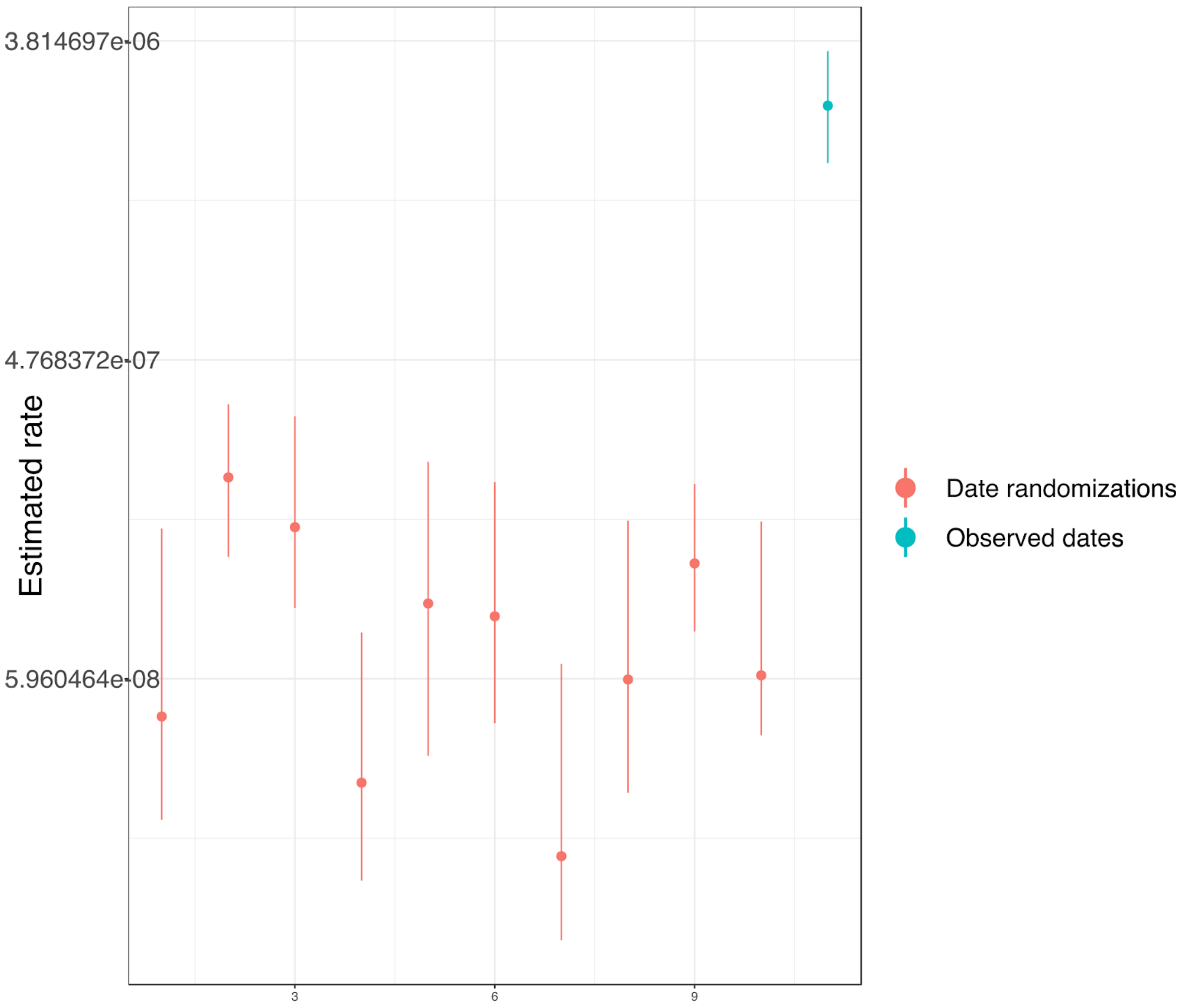

Figure S5. Tip date randomization of time-resolved outbreak clade phylogeny (ST-7827 only). For each of the ten realizations (the red error bars), the date assigned to each sample was randomly selected without replacement from the observed dates. The estimated mutation rates from the randomized date datasets were plotted along with the estimated mutation rate from the observed dates (blue errorbar). None of the ten randomizations had overlapping intervals with the true date, indicating that there was enough temporal signal to estimate the rate.

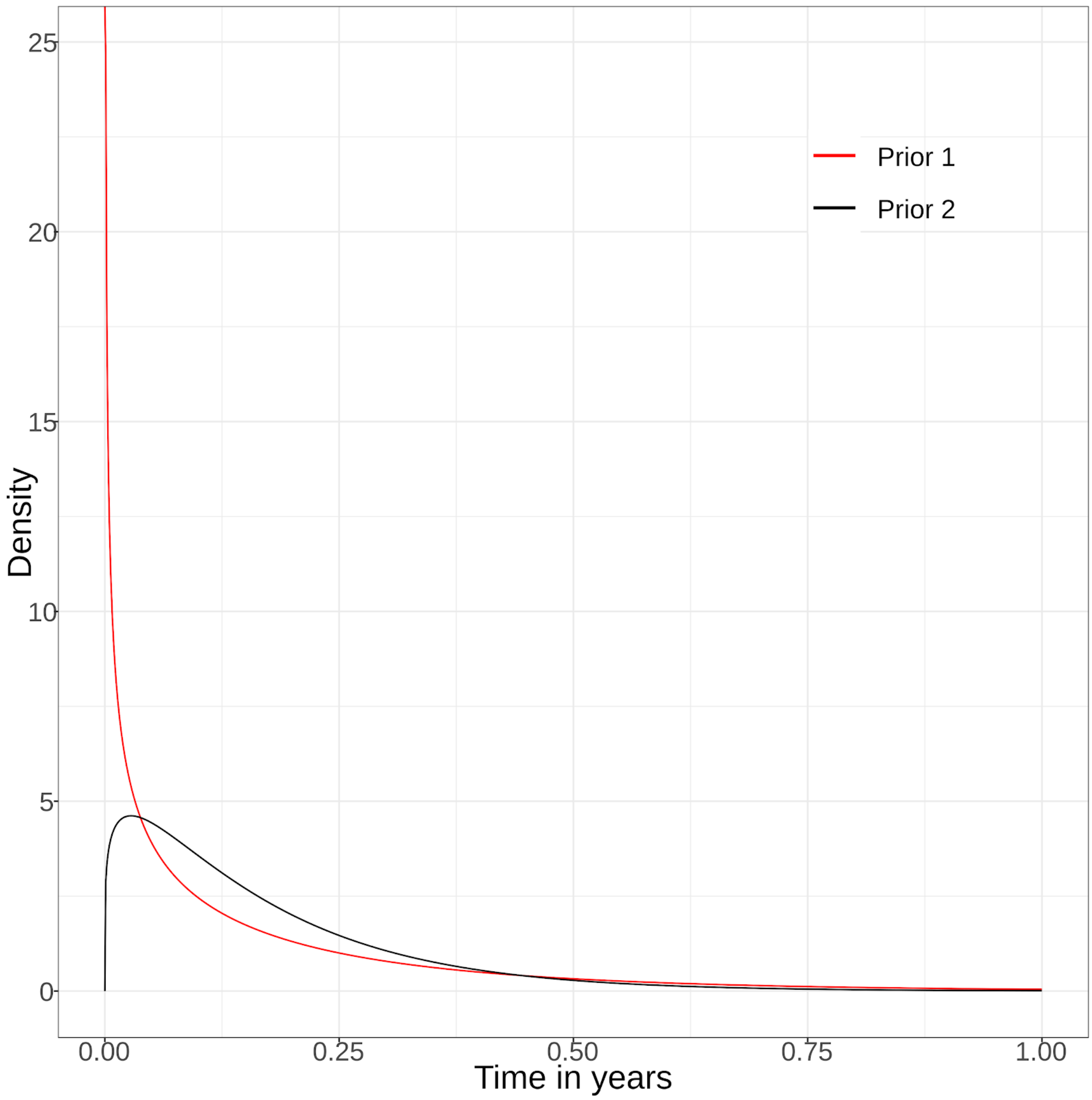

Figure S6. Priors for the generation time and sampling distributions in TransPhylo. The red line shows the prior gamma distribution based on [8] with shape 0,57 and scale 0,3. The black line shows the gamma distribution with shape 1,2 and scale 0,14, which penalizes rapid transmissions and mimics an incubation phase of the pathogen.

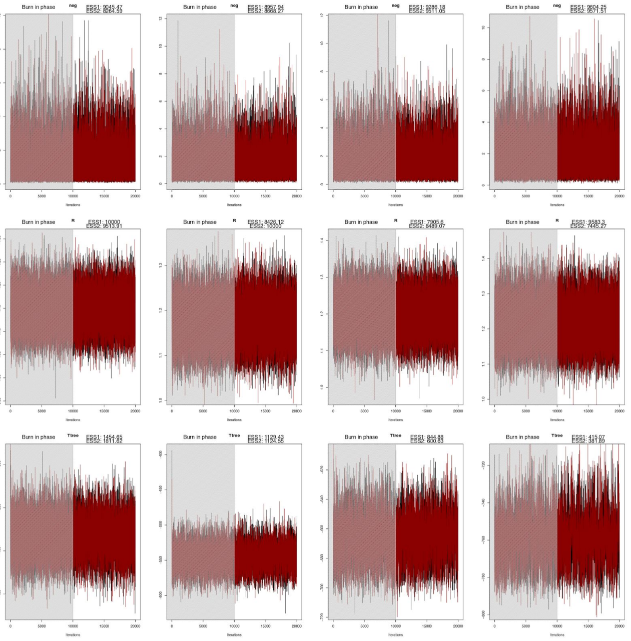

Figure S7. Convergence diagnostics of the Transphylo MCMC-chains for prior 1. The red and black lines show two different realizations of the MCMC for sampling densities 0,2, 0.3, 0.4 and 0,5 in the columns from left to right. The greyed out area shows the iterations discarded as burn-in. The plots in the first row show the chains for within-host effective population size N_{eg}, the second row the reproductive number *R*, and the third row shows the chain for the transmission tree. In all cases, the chains seemed to have achieved sufficient convergence with effective sampling sizes > 200.

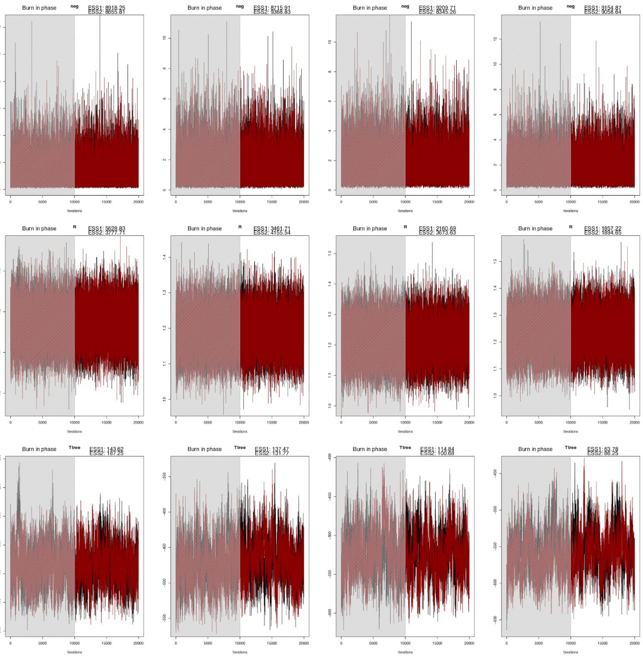

Figure S8. Convergence diagnostics of the Transphylo MCMC-chains for prior 2. The effective sampling size for the transmission-tree (third row) was not perfect (> 50, but < 200), but both chains seemed to converge to the same range, and the estimated parameters from these chains were similar to the estimates from the other chains, indicating proper convergence for these chains as well.

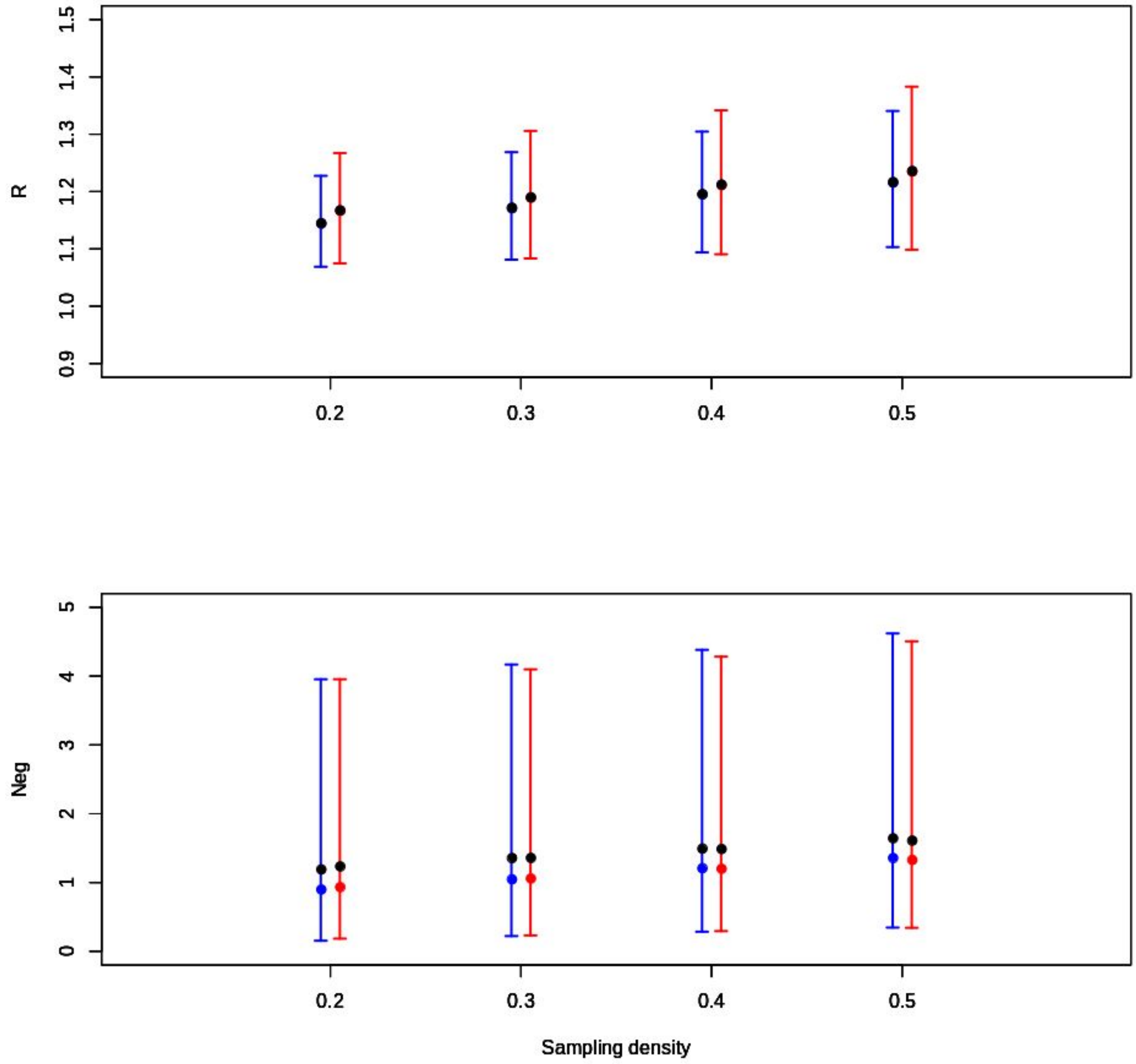

Figure S9. Estimates of the reproductive number *R* and coalescent parameter N_{eg} over different sampling densities. The red and blue error bars show the estimated parameters with prior 1 in red, and prior 2 in blue. The black dot shows the median and the red and blue dots show the mean. Note that the results have overlapping intervals and are quite similar over all the sampling densities and prior distributions considered.

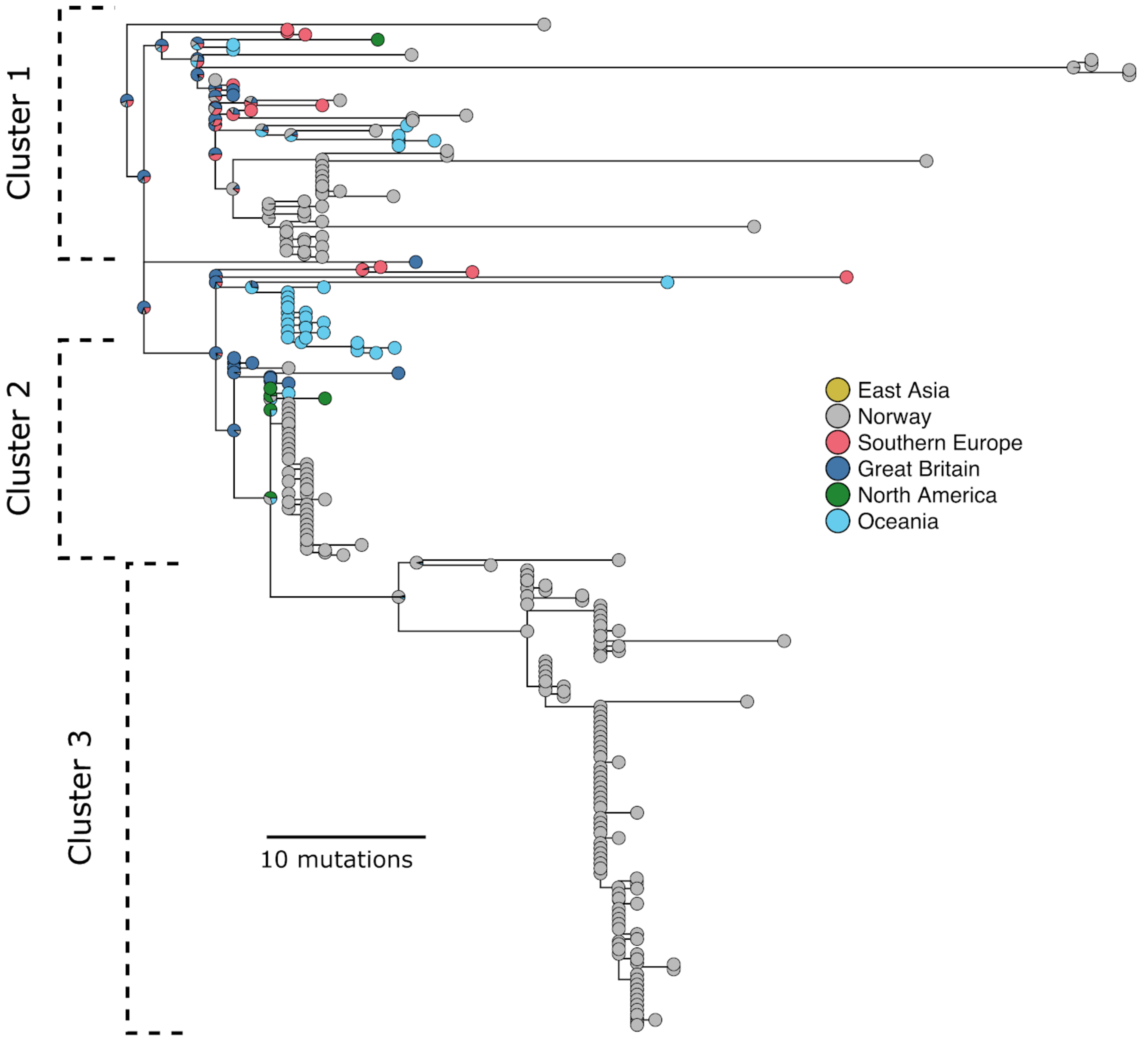

Figure S10. A subset of the phylogeographic mapping in Figure S2. In Cluster 1 there are multiple geographical locations mixed in between the Norwegian samples, indicating that this cluster has been sustained by multiple importation events. Cluster 2 and 3 are geographically restricted to Norway and may have been started by a single introduction event.

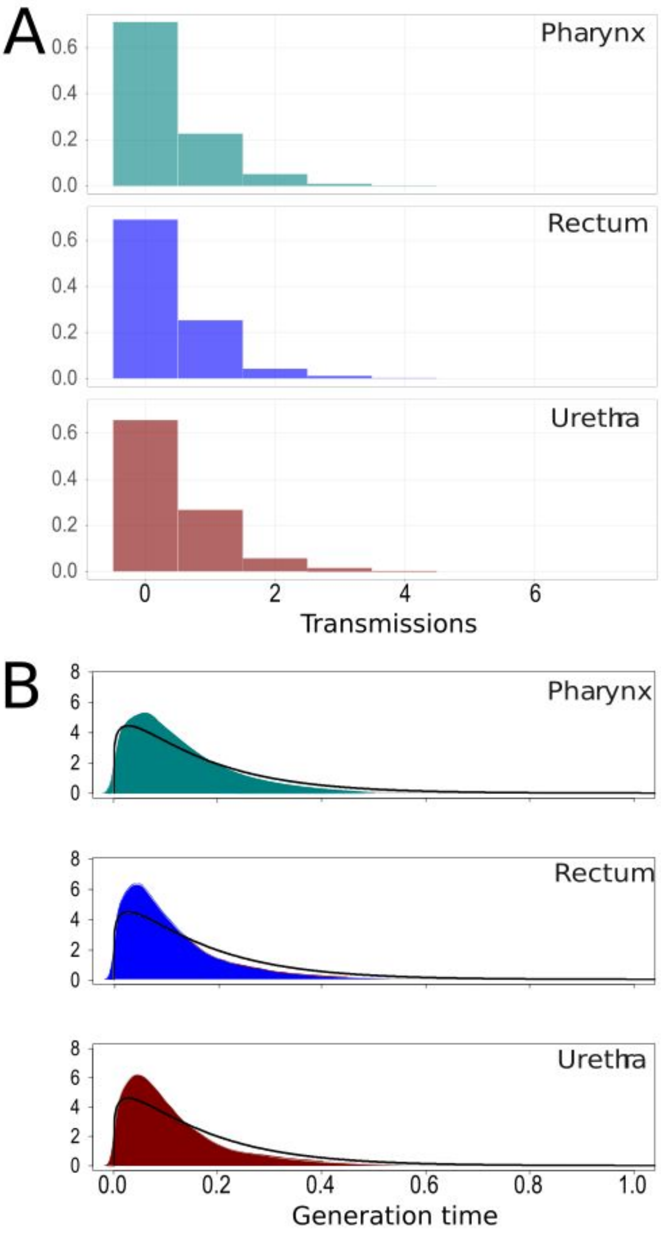

Figure S11. Infection site and transmission properties. A: Histograms of the estimated number of secondary infections caused by a primary infection for different infection sites. B: Density plots of the posterior generation times in years for different infection sites. The black line shows the prior distribution for comparison with the posteriors.

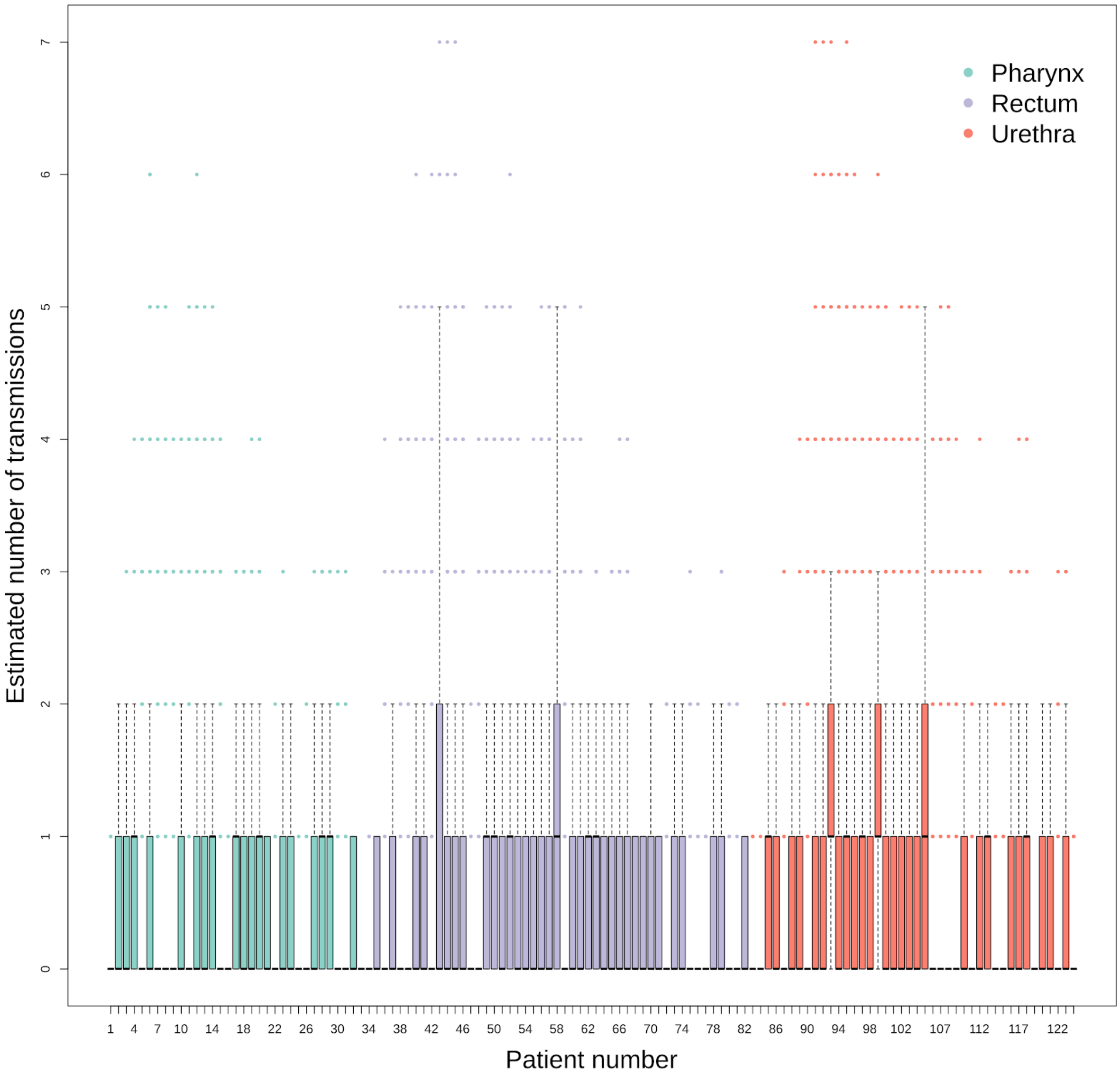

Figure S12. Patient level transmissions. Boxplots of the estimated number of secondary infections caused by a primary infection for each patient over the different transmissions trees. The boxplots are colored and ordered after infection sites. There were no clear differences between the infection sites.

References

1. **Didelot X, Croucher NJ, Bentley SD, Harris SR, Wilson DJ**. Bayesian inference of ancestral dates on bacterial phylogenetic trees. *Nucleic Acids Res* 2018;46:e134.

2. **Hamilton HL, Dillard JP**. Natural transformation of Neisseria gonorrhoeae: from DNA donation to homologous recombination. *Mol Microbiol* 2006;59:376–385.

3. **Bollback JP**. SIMMAP: stochastic character mapping of discrete traits on phylogenies. *BMC Bioinformatics* 2006;7:88.

4. **Revell LJ**. phytools: an R package for phylogenetic comparative biology (and other things). *Methods Ecol Evol* 2012;3:217–223.

5. **Guindon S, Dufayard J-F, Lefort V, Anisimova M, Hordijk W, *et al.*** New algorithms and methods to estimate maximum-likelihood phylogenies: assessing the performance of PhyML 3.0. *Syst Biol* 2010;59:307–321.

6. **Lefort V, Longueville J-E, Gascuel O**. SMS: Smart Model Selection in PhyML. *Mol Biol Evol* 2017;34:2422–2424.

7. **Didelot X, Fraser C, Gardy J, Colijn C**. Genomic Infectious Disease Epidemiology in Partially Sampled and Ongoing Outbreaks. *Mol Biol Evol* 04 01, 2017;34:997–1007.

8. **Whittles LK, White PJ, Didelot X**. A dynamic power-law sexual network model of gonorrhoea outbreaks. *PLoS Comput Biol* 2019;15:e1006748.
